# The Association between Use of Benzodiazepine Receptor Agonists and the Risk of Obstructive Sleep Apnea: A Nationwide Population-Based Nested Case-Control Study

**DOI:** 10.3390/ijerph18189720

**Published:** 2021-09-15

**Authors:** Tien-Wei Hsu, Hsiu-Min Chen, Tien-Yu Chen, Che-Sheng Chu, Chih-Chuan Pan

**Affiliations:** 1 Department of Psychiatry, Kaohsiung Veterans General Hospital, Kaohsiung 81362, Taiwan; twhsu@vghks.gov.tw; 2 Department of Health-Business Administration, Fooyin University, Kaohsiung 83130, Taiwan; cshowme@gmail.com; 3 Department of Psychiatry, Tri-Service General Hospital, Taipei 11490, Taiwan; verducciwol@gmail.com; 4 Institute of Brain Science, National Yang-Ming University, Taipei 11221, Taiwan; 5 Center for Geriatric and Gerontology, Kaohsiung Veterans General Hospital, Kaohsiung 81362, Taiwan; 6 Non-Invasive Neuromodulation Consortium for Mental Disorders, Society of Psychophysiology, Taipei 11221, Taiwan; 7 Graduate Institute of Medicine, College of Medicine, Kaohsiung Medical University, Kaohsiung 80737, Taiwan

**Keywords:** benzodiazepine receptor agonists, nested case-control study, obstructive sleep apnea, benzodiazepine, Z-drugs

## Abstract

Obstructive sleep apnea (OSA) is characterized by recurrent upper airway collapse. Benzodiazepine receptor agonists (BZRAs) are associated with pharyngeal muscle relaxation, increased apnea duration, and hypoxia, which might worsen OSA. This study aimed to examine the association between the use of BZRAs and the risk of OSA. The study was conducted using data from the National Health Insurance Database of Taiwan between 2002 and 2011. We only included new users who were never exposed to any BZRAs and identified 1848 participants with OSA, and 1848 matched controls. A logistic regression model was used to determine the association between the use of BZRAs and the development of OSA. BZRA exposure was divided into usage patterns, dosage, duration, and pharmacokinetic class. We found an increased risk of OSA in current users and recent past users compared with distant past users. Patients with a higher cumulative dose of BZRAs were more likely to develop OSA compared to those with a lower cumulative dose. We found an increased risk of OSA in patients treated with BZRAs, especially for current users and those with higher cumulative doses. A reduced risk of OSA was found in Z-drug users compared with benzodiazepine users.

## 1. Introduction

Obstructive sleep apnea (OSA) is characterized by sleep-related recurrent upper airway collapse, resulting in oxygen desaturation during sleep [1]. The prevalence of OSA has increased, affecting 9% to 38% of the adult population, with a higher occurrence rate in men [2]. Evidence has suggested several screening tools that could be used to identify OSA, including the Berlin Questionnaire, modified Mallampati scores, pulse oximetry readings, and upper airway volume data obtained from Cone Beam Computed Tomography [3]. The severity of OSA is determined by the apnea-hypopnea index (AHI) which is defined as the number of times an individual has apnea or hypopnea for one night, divided by the hours of sleep measured by polysomnography (PSG) [4]. Several adverse medical outcomes are associated with OSA, including myocardial infarction, metabolic disorders, stroke, hypertension, and impaired cognitive function [5,6]. One of the most significant risk factors is obesity [1]; others include advancing age [1], male sex [1], and the potential risk factor of using sedative medications [7].

Around 39–54.9% of insomnia cases have been reported in patients with OSA [8]. A two-year prospective study reported a high co-occurrence of insomnia and sleep-disordered breathing symptoms in community-based primary care clinics [9]. Benzodiazepine receptor agonists (BZRAs), including benzodiazepines (BZDs), and the drugs zopiclone, zolpidem, and eszopiclone (referred to as “Z-drugs”), are commonly prescribed for insomnia. However, BZRAs are traditionally not recommended in patients with OSA due to the concern of worsening OSA via pharyngeal muscle relaxation, increased apnea duration, and hypoxia [10,11]. In recent years, growing evidence has demonstrated that Z-drugs do not elevate the arousal threshold [12,13,14,15].

The variable effect of Z-drugs and BZDs on the risk of OSA might be explained by several hypotheses. First, BZDs might reduce upper airway muscle activity, leading to a reduction in respiratory flow and oxygen desaturation, and in turn, resulting in an increased AHI and exacerbation of hypoxia and hypercapnia [11,16,17,18,19,20]. Second, BZDs suppress central ventilatory drive and respiratory muscle motor neurons by binding to BZD receptors to activate the GABA system in motor neurons [21]. Third, the administration of the standard dose of Z-drugs is effective in improving objective sleep quality without the adverse effect of increasing AHI, worsening pulse oximetry, or arousal threshold [22,23]. Furthermore, a single administration of 3 mg eszopiclone decreased the AHI compared to the placebo [14]. Taken together, BZDs have a greater effect on respiratory depression than Z-drugs [24,25] and Z-drugs have less prominent muscle-relaxant effects than BZDs via different receptor affinity/binding sites of the GABA receptor [12,22]. On the contrary, another cohort study revealed that concomitant BZDs and opioids decrease AHI, respiratory arousal index and sleep apnea risk compared with sole opioid users [26]. Therefore, future studies to examine the effect of BZDs and Z-drugs on the risk of OSA are needed.

The BZRA users are more frequently associated with older age, female sex, mental disorders, depression, obesity, metabolic syndrome, diabetes, and hypertension [27,28], which may possibly lead to the development of OSA.

So far, few published studies have explored the role of BZRAs in the risk of OSA development. Given the existing evidence regarding the converse effect of BZDs and Z-drugs on PSG among patients with OSA [10,11], we hypothesized that the use of BZDs and Z-drugs was associated with a different degree of impact on the risk of OSA. We further hypothesized that the duration, dose-response, and pharmacokinetic class of BZRAs might also influence the risk of OSA.

## 2. Materials and Methods

### 2.1. Data Source

The National Health Insurance Research Database (NHIRD) in Taiwan was launched in March 1995, and covered more than 99% of Taiwan’s population by the end of 2010 [29]. As it is one of the largest insurance databases in the world, it contains all claims data from both inpatients and outpatients from Taiwan and has provided valuable information in several epidemiological studies [29,30,31]. Clinical diagnoses were coded according to the International Classification of Diseases, 9th Revision, Clinical Modification (ICD-9-CM). This study was based on the Longitudinal Health Insurance Database 2000 (LHID 2000), which contains 1,000,000 individuals randomly selected from the NHI Registry for Beneficiaries in 2000, accounting for approximately 5% of the total population in Taiwan. The LHID contains claims data covering all medical procedures and prescriptions dating from January 1996 to December 2011 (http://nhird.nhri.org.tw/date_01_en.html accessed on 12 March 2021). This study was conducted in accordance with the Declaration of Helsinki and relevant guidelines, and approved by the institutional review board of the Kaohsiung Veterans General Hospital, Kaohsiung, Taiwan (IRB: VGHKS15-EM10-02).

### 2.2. Study Design

Patients prescribed with BZRAs are more likely to receive PSG, and therefore be diagnosed with OSA [32]. Thus, we restricted our population to patients who were never exposed to BZRAs to reduce unmeasured confounding and selection bias. In addition, based on previous studies [33,34], we adopted new-user design, by which the whole studied populations including both experimental group and control group were new users of BZRAs to reduce the risk of confounding by indication of disease, severity, and unmeasured clinical characteristics.

We defined the new users (cohort entry date) as participants receiving the newly prescribed medication or dispensing event of the BZRAs on record between 2002 and 2011. The LHID database was established since 2000, from which we collected data between 2002 and 2011. This ensured that the new users did not have BZRA prescriptions at least in the preceding two years as a washed-out period.

The study outcome was to determine the incidence of OSA (ICD-9-CM code: 327.23, 780.51, 780.53, or 780.57) among new users of BZRAs during the follow-up period. To increase diagnostic validity, new incident cases of OSA should undergo PSG (17008A, 17008B) within one year before or after the diagnosis of OSA was made [35]. The date of the first diagnosis of OSA was defined as the index date for that patient. The index date of each control was assigned as equal to the index date of the respective matched case. Previous studies have demonstrated that the validity and accuracy of the NHIRD claimed data with respect to the medical records of PSG was 87% [36].

New users of BZRAs who had been diagnosed with OSA (defined by index date) in the follow-up period were selected as the OSA group. New users of BZRAs without a diagnosis of OSA in the whole follow-up period were selected as the control group. Each control was selected from the same database and matched by age, sex, and index date at a ratio of 1:1.

We defined three windows of exposure to individual BZRA drugs: current users (at least one prescription within 90 days before the index date), recent past users (latest prescription within 91–365 days before the index date), and distant past users as the reference group (no prescription within 365 days before the index date). We used 90- and 365-day cut-off values, as these cut-offs have been adopted in previous studies [34].

### 2.3. Study Population

**Figure 1:**
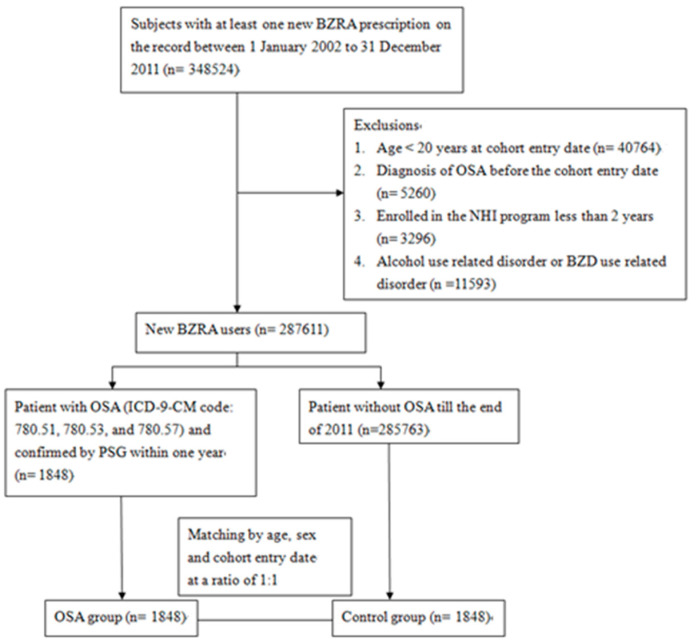
Flow chart of selection of study subjects from Taiwan National Health Insurance Database. ICD-9-CM = International Classification of Disease, Ninth Revision, Clinical Modification. BZRA = benzodiazepine receptor agonist. NHI = National Health Insurance. OSA = obstructive sleep apnea. PSG = polysomnography.

We conducted this case-control study by first analyzing the NHIRD data from 2002 to 2011. We assembled a cohort of NHI participants aged ≥ 20 who corresponded to the definition of new users of BZRAs between 2002 and 2011. The exclusion criteria were as follows: (1) Participants aged < 20 years old; (2) those enrolled in the NHIRD for less than two years prior to the cohort entry date; (3) participants who had been diagnosed with OSA (ICD-9-CM codes 780.51, 780.53, and 780.57) before the cohort entry date; (4) alcohol abusers, who had been diagnosed with alcohol poisoning (ICD-9-CM codes 980.0, 980.1, 980.9), excessive blood level of alcohol (ICD-9-CM codes 790.3), alcoholic psychosis (ICD-9-CM codes 291), alcohol abuse (ICD-9-CM codes 305, 303) and alcoholic liver disease (ICD-9-CM codes 571.0~571.3); (5) hypnotics abusers, who had been diagnosed with sedative, hypnotic or anxiolytic abuse (ICD-9-CM codes 304.1, 305.4) and hypnotics intoxication (ICD-9-CM codes 968.4, 969.4). The participants were followed up from 1 January 2002 to 31 December 2011, or until the diagnosis of OSA was made, or participants disenrolled from the NHIRD. Figure 1 presents a detailed flow chart regarding participant selection.

### 2.4. Exposure to BZRAs

Data on BZRAs drugs use were obtained from prescription files from the NHIRD. The BZRAs dose is the cumulative number of the defined daily doses (DDDs). Drugs were classified according to the Anatomical Therapeutic Chemical (ATC) classification system [37]. The ATC system classifies BZRA into three groups based on the ATC code: benzodiazepine anxiolytics (N05BA), benzodiazepine hypnotics (N05CD), and non-benzodiazepine hypnotics (N05CF). Although clonazepam was classified as an antiepileptic drug (N03AE) in the ATC system, and it is widely used as an anxiolytic drug in Taiwan. Thus, we included clonazepam as an anxiolytic agent in our study. The daily dose is based on the international standard DDDs [38]. The cumulative BZRAs DDDs was calculated by dividing the BZRA dosage during the study period by the DDDs. Cumulative BZRAs DDDs were classified into three subgroups: (a) DDDs ≤ 28, (b) DDDs = 29–90, and (c) DDDs > 90 [33].

We further considered the duration of action of BZRAs. Thus, we categorized BZDs into three groups according to their elimination half-life as ultra-short acting (<5 h) (triazolam, midazolam, and brotizolam), short-intermediate acting (5–24 h) (oxazepam, lorazepam, bromazepam, alprazolam, fludiazepam, nitrazepam, flunitrazepam, estazolam, and lormetazepam), and long-acting (>24 h) (diazepam, chlordiazepoxide, medazepam, clorazepate dipotassium, clobazam, nordazepam, oxazolam, flurazepam, and clonazepam) [39,40]. Additionally, Z-drugs are separately classified as ultra-short-acting due to their different mechanisms of action on gamma aminobutyric acid (GABA) receptors [41].

### 2.5. Primary and Secondary Outcomes

We defined the primary outcome as the risk of OSA development among different BZRA exposure windows (current users, recent past users, and distant past users) and different classes of BZRA use (BZDs alone, Z-drugs alone, and combined with BZDs and Z-drugs). The secondary outcomes were the effect of (1) a cumulative dose of BZRAs, (2) the number of combinations of BZRAs, and (3) the pharmacokinetic class of BZRAs on the risk of OSA development.

### 2.6. Potential Confounding Factors

The confounding variables of this study were physical illness and psychotropic drugs, which were assessed in the year preceding the cohort entry date. Physical illness included cerebrovascular disease (ICD-9-CM codes: 430*–438*)(*present all ICD codes title starting with this code), hypertension (ICD-9-CM codes: 401*–405*), diabetes (ICD-9-CM codes: 250*), ischemic heart disease (ICD-9-CM codes: 410*–415), hyperlipidemia (ICD-9-CM codes: 272), chronic obstructive pulmonary disease (ICD-9-CM codes: 490*–496*), congestive heart failure (ICD-9-CM codes 428, 398.91, and 402.x1), chronic kidney disease (ICD-9-CM codes 582, 583, 585, 586, and 588), hypothyroidism (ICD-9-CM code 244), pneumonia (ICD-9-CM codes 480*–486*), lung abscesses (ICD-9-CM code 513*), and empyema (ICD-9-CM codes 510*). Psychotropic drugs included antipsychotics (ATC codes: N05AA, N05AB, N05AC, N05AD, N05AE, N05AF, N05AG, N05AH, N05AL, and N05AX), antidepressants (ATC code: N06A), anti-epilepsy (ATC code: N03A, except N03AC), and opioids (ATC codes: N01AH, N02AG, N02AJ, N02AX).

### 2.7. Statistical Analysis

Chi-square tests and Student’s *t*-tests were performed to compare categorical and continuous variables in addition to demographic and clinical characteristics between the case and control groups. A conditional logistic regression analysis was used to examine the effect of BZRAs usage on the risk of OSA development and to control for potential confounding factors. A multivariate regression analysis was used to estimate the effect of (1) BZRA drug use (current/recent past users versus distant past users); (2) classes of BZRAs (Z-drugs alone or combined with BZDs vs. BZDs alone); and (3) pharmacokinetic classes of BZRAs (ultra-short, short-intermediate, and long-acting agents vs. Z-drugs) on the risk of OSA development. All statistical tests were two-sided, conducted at a significance level of *p* values ≤ 0.05, and the odds ratio (OR) and 95% confidence intervals (CIs) reported. All analyses were performed using SAS version 9.2 (SAS Institute, Cary, NC, USA) and SPSS version 20.0 for Windows (SPSS, Chicago, IL, USA).

## 3. Results

### 3.1. Clinical Characteristics of the Study Population

Data from a total of 3696 enrolled participants (1848 patients in the OSA-group and 1848 patients in the non-OSA group) were analyzed. Table 1 shows the distribution of demographic characteristics, comorbid physical illness, and prescribed medications between the two groups. The mean age of the OSA and non-OSA groups were 42.5 ± 12.6 and 42.5 ± 12.7 years, respectively. Patients with OSA had significantly higher incidence of hypertension, ischemic heart disease, hyperlipidemia, and chronic obstructive pulmonary disease (Table 1).

### 3.2. Primary Outcome: Exposure to and Use of BZRAs and Risk of OSA Development

Regarding OSA development associated with BZRA exposure, we found significantly increased risk in current (adjusted odds ratio [aOR]: 4.02, 95% CIs: 3.36–4.82) and recent past BZRA users (aOR: 1.45, 95% CIs: 1.17–1.80) versus distant past users after adjustment. With regard to the subgroup analysis of BZRAs, we found a significantly reduced risk of OSA in Z-drug users (aOR: 0.66, 95% CIs: 0.44–0.99) versus BZD users, whereas a significantly increased risk of OSA was found in concomitant BZD and Z-drug users (aOR: 1.63, 95% CIs: 1.39–1.90) compared to BZD-alone users. In addition, we focused on the risk of the BZRA subgroups in development of OSA. In terms of current users, patients who used both BZDs and Z-drugs were more likely to develop OSA (aOR: 1.90, 95% CIs: 1.38–2.62) compared with those receiving only BZDs. No significant difference in the risk of OSA development was found in the status of recent past and distant past users (Table 2).

### 3.3. Secondary Outcomes: Risk Association between Several BZRAs and Cumulative Dosage in OSA Development

Compared with BZRA monotherapy, higher numbers of concomitant BZRAs were significantly associated with an increased risk of OSA (number = 2, aOR: 1.42, 95% CIs: 1.19–1.70; number = 3, aOR: 2.04, 95% CIs: 1.65–2.52; number = 4, aOR: 2.52, 95% CIs: 2.06–3.08). Compared to the patients exposed to BZRAs DDDs ≤ 28, a significantly increased risk of OSA was found in subgroups with cumulative BZRAs DDDs = 29–90 (aOR: 1.39, 95% CIs: 1.15–1.68) and DDDs > 90 (aOR, 1.60; 95% CIs, 1.33–1.93) (Table 3).

### 3.4. Secondary Outcomes: Pharmacokinetic Property Risk Association of BZRAs in OSA Development

We classified BZRAs based on their pharmacokinetic properties into four classes: Z-drugs, ultrashort-acting, short-immediate, and long-acting agents. Exposure to more than one class of BZRAs was excluded (*n* = 827), resulting in a final of 2869 participants. No significant difference was found between different classes and risk of OSA development (Table 4).

## 4. Discussion

To the best of our knowledge, this is the first study using a nationwide, population-based dataset to explore the association between the use of BZRAs and the risk of developing OSA. The main findings can be summarized as follows: (1) the use of BZRAs was significantly associated with an increased risk of OSA development; (2) the use of Z-drugs had significantly reduced risk of OSA development compared with the use of BZDs; (3) current and recent past users of BZRAs had an increased risk of OSA development compared with distant past users; (4) a dose-response effect of increased risk of OSA was observed in patients using BZRAs; and (5) higher numbers of concomitant BZRAs were significantly associated with an increased risk of OSA compared with BZRA monotherapy.

A retrospective analysis of the Danish database, analyzing 38,735 patients with OSA and 75,941 controls, found that psychotropic medication use including BZDs and Z-drugs was more frequent in patients with OSA [42]. The present study extended the prior findings by showing that BZRA use was associated with an elevated risk of OSA. Furthermore, we found that Z-drug users had a significantly reduced risk of OSA development compared with BZD users.

The risk of OSA development was most profound among current BZRAs users (within 90 days), followed by recent past users (91 to 365 days) compared to distant past users (over 365 days). The development of OSA might be a long-term and chronic process. The deteriorating effect of BZRAs on the risk of OSA appears within one year of use of BZRAs, usually within the first three months. This present study is the first to discuss the durational effect of BZRAs on the risk of OSA and the results are preliminary, requiring further studies to confirm the findings.

A higher cumulative dose or number of concomitant BZRAs increased the risk of OSA. Higher cumulative BZRAs DDDs showed a significantly higher risk for developing OSA compared to lower cumulative doses. These findings are consistent with previous studies, showing that the adverse effects of BZRAs in the respiratory system may vary, depending on the dosage patterns [24]. However, past studies [14,17,18,19] examining the relationship between OSA and BZRAs were short-term, single-dose, and standard-dose trials. Therefore, they could not reflect the actual clinical situations presenting with poly-pharmacy, long-term use, or higher doses.

The present study found no significant difference between different BZRAs based on their pharmacokinetic properties and risk of OSA development. Therefore, the duration of action of BZRAs might play a little role in the development of OSA. However, owing to the inadequate sample size of each pharmacokinetic property group, firm conclusions were not established, thus no significant difference was found between the different classes and the risk of OSA development. Future studies are thus warranted to confirm this finding.

The strengths of the study include its use in a nationwide representative population with large sample sizes, which minimized selection bias. Further, the exposure status, dose, duration, and class of BZRAs were comprehensively assessed. However, several limitations of this study should be addressed. First, several potential confounding factors, such as smoking habits, alcohol consumption, neck circumference, lifestyle, and family history, were not available in the NHIRD. Importantly, due to the limitation of NHIRD, we could not obtain body mass index (BMI) data, which is one of the most crucial confounding factors of OSA [43]. We have tried to use the diagnosis of obesity (ICD-9-CM code: 278) instead of BMI. Unfortunately, we could only identify 11 patients diagnosed as obese in the study population. Under the impression of an under-record of the diagnosis of obesity, we did not use obesity as confounding factor in this study. Indeed, specific populations, especially obese individuals, are more likely to use sedative medications, being one of the major risk factors of developing OSA [44]. Therefore, we used a new-user design to limit our study population to new users of BZRAs, which could be considered as a more homogenous population. Such a study design could alleviate the bias of BMI and other potential confounding factors. Secondly, the causal relationship between BZRAs and the development of OSA cannot be fully elucidated, as we adopted a nested case-control study design. Thirdly, medication exposure was based on prescription records, and thus adherence was not possible to assess using the claims databases. Fourth, all patients with a diagnosis of OSA were retrieved from claims data using ICD-9-CM codes. We could not calculate those which were under-diagnosed. Referral bias could not be avoided. Finally, the patients of the study were of the Chinese ethnic population. Hence, it is difficult to generalize the present findings to different ethnic groups.

## 5. Conclusions

We found an increased risk of OSA among patients treated with BZRAs, especially in higher cumulative doses and higher numbers of concomitant BZRAs. Moreover, BZDs seem to have a greater risk than Z-drugs of developing OSA. The results of the present study should prompt clinicians to prescribe hypnotics at the lowest effective dose, thus avoiding prolonged and combination prescriptions. Although the study was not a causal relationship study, the findings might guide clinicians and patients in shared decision-making for treatment.

## Figures and Tables

**Table 1:** Demographics and clinical characteristics of patients in the non-OSA and OSA groups (*n* = 3696).

Variables	OSA (*n* = 1848)	Non-OSA (*n* = 1848)	*p* Value
**Demographics**			
Age, years, mean ± SD	42.5 ± 12.6	42.5 ± 12.7	0.948
Sex, male, %	1336 (72.3)	1336(72.3)	>0.999
**Comorbidity**			
Cerebrovascular disease, %	32 (1.7)	30 (1.6)	0.898
Hypertension, %	323 (17.5)	215 (11.6)	<0.001
Diabetes, %	101 (5.5)	81 (4.4)	0.148
Ischemia heart disease, %	111 (6.0)	55 (3.0)	<0.001
Hyperlipidemia, %	167 (9.0)	107 (5.8)	<0.001
Chronic obstructive pulmonary disease, %	135 (7.3)	82 (4.4)	<0.001
Congestive heart failure, %	19 (1.0)	12 (0.6)	0.279
Chronic kidney disease, %	19 (1.0)	28 (1.5)	0.240
Pneumonia, %	31 (1.7)	24 (1.3)	0.415
**Sedative medication, %**			
Antipsychotics, %	606 (32.8)	560 (30.3)	0.111
Antidepressant, %	496 (26.8)	327 (17.7)	<0.001
Anti-epilepsy, %	374 (20.2)	253 (13.7)	<0.001
Opioid, %	168 (9.1)	118 (6.4)	0.003

Abbreviations: OSA: obstructive sleep apnea; SD: standard deviation.

**Table 2:** Use of BZRAs and risk of OSA development (*n* = 3696).

Variables	OSA (*n* = 1848)	Non-OSA (*n* = 1848)	Crude OR	95% CI for Crude OR	aOR *	95% CI for aOR
**Overall sample**						
Distant past use (>365 days)	922 (49.9)	1376 (74.5)	1.00	Reference	1.00	Reference
Current use (≤90 days)	712 (38.5)	251 (13.6)	4.23	(3.59–5.00)	4.02	(3.36–4.82)
Recent past use (91–365 days)	214 (11.6)	221 (12.0)	1.45	(1.18–1.78)	1.45	(1.17–1.80)
**Class of BZRAs**						
BZDs	1118 (60.5)	1347 (72.9)	1.00	Reference	1.00	Reference
Z-drugs	39 (2.1)	71 (3.8)	0.66	(0.44–0.99)	0.66	(0.44–0.99)
BZDs + Z-drugs	691 (37.4)	430 (23.3)	1.94	(1.68–2.24)	1.63	(1.39–1.90)
**Distant past use**						
BZDs	752 (81.6)	1085 (78.9)	1.00	Reference	1.00	Reference
Z-drugs	33 (3.6)	65 (4.7)	0.73	(0.48–1.13)	0.73	(0.47–1.13)
BZDs + Z-drugs	137 (14.9)	226 (16.4)	0.88	(0.69–1.10)	0.77	(0.60–1.00)
**Current use**						
BZDs	226 (31.7)	122 (48.6)	1.00	Reference	1.00	Reference
Z-drugs	5 (0.7)	3 (1.2)	0.90	(0.21–3.83)	1.02	(0.21–4.90)
BZDs + Z-drugs	481 (67.6)	126 (50.2)	2.06	(1.53–2.77)	1.90	(1.38–2.62)
**Recent past use**						
BZDs	140 (65.4)	140 (63.3)	1.00	Reference	1.00	Reference
Z-drugs	1 (0.5)	3 (1.4)	0.33	(0.03–3.24)	0.36	(0.04–3.69)
BZDs + Z-drugs	73 (34.1)	78 (35.3)	0.94	(0.63–1.34)	0.90	(0.58–1.41)

* Adjustment for hypertension, ischemia heart disease, hyperlipidemia, chronic obstructive pulmonary disease, antipsychotic, antidepressant, anticonvulsant, and opioid. Abbreviations: aOR: adjusted odds ration; BZDs: benzodiazepines; BZRAs: benzodiazepine receptor agonists; CI: confidence interval; OSA: obstructive sleep apnea; SD: standard deviation.

**Table 3:** Association between several kinds and dosages of BZRAs and risk of OSA development (*n* = 3696).

Variables	OSA (*n* = 1848)	Non-OSA (*n* = 1848)	Crude OR	95% CI for Crude OR	aOR *	95% CI for aOR
**BZRAs number**						
number = 1	533 (28.8)	841 (45.5)	1.00	Reference	1.00	Reference
number = 2	431 (23.3)	451 (24.4)	1.51	(1.27–1.79)	1.42	(1.19–1.70)
number = 3	312 (16.9)	234 (12.7)	2.10	(1.72–2.57)	2.04	(1.65–2.52)
number ≥ 4	572 (31.0)	322 (17.4)	2.80	(2.34–3.34)	2.52	(2.06–3.08)
**BZRAs cDDDs**						
≤28	980 (53.0)	1240 (67.1)	1.00	Reference	1.00	Reference
29–90	337 (18.2)	270 (14.6)	1.58	(1.32–1.89)	1.39	(1.15–1.68)
>90	531 (28.7)	338 (18.3)	1.99	(1.69–2.33)	1.60	(1.33–1.93)

* aOR Adjustment for hypertension, ischemia heart disease, hyperlipidemia, chronic obstructive pulmonary disease, antipsychotic, antidepressant, anticonvulsant, and opioid. Abbreviations: aOR: adjusted odds ratio; BZRAs: benzodiazepine receptor agonists; cDDD: cumulative defined daily dose; CI: confidence interval; OSA: obstructive sleep apnea.

**Table 4:** Association of BZRAs based on different pharmacokinetic properties and risk of OSA development (*n* = 2869, excluded 827 subjects who had an exposure of more than one class).

Variables	OSA (*n* = 1316)	Non-OSA (*n* = 1553)	Crude OR	95% CI for Crude OR	aOR *	95% CI for aOR
**Pharmacokinetic class ^#^**						
Z-drugs	249 (18.9)	240 (15.5)	1.00	Reference	1.00	Reference
Ultrashort-acting	47 (3.6)	39 (2.5)	1.16	(0.73–1.84)	1.12	(0.70–1.81)
Short-intermediate-acting	333 (25.3)	438 (28.2)	0.73	(0.58–0.92)	0.81	(0.64–1.03)
Long-acting	687 (52.2)	836 (53.8)	0.79	(0.65–0.97)	0.96	(0.77–1.19)

* aOR Adjustment for social economic status, hospital level, hospital area, hypertension, ischemia heart disease, hyperlipidemia, chronic obstructive pulmonary disease, antipsychotic, antidepressant, anticonvulsant, and opioid. ^#^ Z-drug: zolpiclone, zolpidem and zaleplon; ultrashort-acting agents: triazolam, midazolam and brotizolam; short-intermediate-acting agents: oxazepam, lorazepam, bromazepam, alprazolam, fludiazepam, nitrazepam, flunitrazepam, estazolam and lormetazepam; long-acting agents: diazepam, chlordiazepoxide, medazepam, clorazepate dipotassium, clobazam, nordazepam, oxazolam, flurazepam and clonazepam. Abbreviations: aOR: adjusted odds ratio; BZRAs: benzodiazepine receptor agonists; CIs: confidence intervals; OSA: obstructive sleep apnea.

## Data Availability

The NHIRD was released and audited by the Department of Health and Bureau of the NHI Program for scientific research (https://nhird.nhri.org.tw/ accessed on 12 March 2021). The NHIRD can be obtained through the formal application regulated by the Department of Health and Bureau of the NHI Program.
